# Septic Bleeding of the Common Carotid Artery Following Total Thyroidectomy: An Atypical Complication

**DOI:** 10.1155/2010/953282

**Published:** 2010-03-07

**Authors:** T. Jamaan, J. Raedecke, C. Kayser, K. D. Rueckauer, O. Thomusch

**Affiliations:** Department of General Surgery, University of Freiburg, Hugstetter Str. 55, 79106 Freiburg, Germany

## Abstract

Septic rupture of the common carotid artery following total thyroidectomy may rapidly lead to exsanguination. We present a case report of a 16-year-old girl, diagnosed with a questionable thyroglossal duct cyst. Following the initial operative intervention with local excision of the cyst including resection of the medial part of the hyoid bone, pathology revealed papillary carcinoma. Thus secondary total thyroidectomy with locoregional lymphadenectomy was performed. One week later, a wound infection developed, necessitating lavage and drainage. On the 8th postoperative day, a dramatic bleeding of the right common carotid artery occurred. To our knowledge, this is the first reported case in the literature with a septic bleeding of the common carotid artery following total thyroidectomy after one week.

## 1. Case Report

A 16-year-old girl had surgery with the differential diagnosis of a median cervical cyst or thyroglossal duct cyst. The family history was otherwise unremarkable. Histopathology showed a papillary thyroid carcinoma. An evaluation with magnetic resonance imaging and radioisotope scanning of the thyroid gland as well as careful examination with regard to suspicious lymph nodes or metastases revealed no signs of further disease. Thus the girl underwent a reoperation four weeks later. The second operation appeared to be difficult at the upper thyroid lobe pole due to tight granulation tissue and scars. During the operation, magnifying, glasses were used. Both recurrent laryngeal nerves and all four parathyroid glands were identified. A total thyroidectomy with systematic dissection of the cervicocentral lymph node compartment was performed. One of the four parathyroid glands was autotransplanted into the left sternocleidomastoideus muscle. During the first day after the operation, the girl had symptomatic hypocalcemia, requiring intravenous substitution of calcium. On the fourth postoperative day, the girl developed a local erythema of the Kocher incision with fever and increased inflammation parameters. After opening of the wound, a small amount of pus was evacuated. Extensive lavage was performed daily. Fever and inflammation parameters were decreasing. At day 8 after the operation, while the girl was in good condition and the wound was unsuspicious, a sudden severe bleeding from the opened neck wound occurred spontaneously. Under manual compression of the wound, the girl was transferred to the operating room rapidly. During disinfection, the hemorrhage had ceased by the compression. Intraoperatively, the tissue was clean except fibrin layers on the muscles and the vessel walls. After careful separation of the planes in the depth of the wound, a severe bleeding developed. Digital compression and step-by-step exploration of the main vessels demonstrated a defect in the median wall of the right common carotid artery in the caudal third, about 6 × 3 mm in size with frayed margins. The distance from this defect to the origin of the carotid artery was scanty for the placement of a vascular clamp. Resection of the faulty artery segment was performed in an extension of 4 cm. An autograft from the saphenous vein was taken at the left proximal thigh. Blood flow could be detected to be 600 mL/min within the graft after reconstruction. In addition, a clamping time of the artery of 21 minutes was necessarily. As well as intra- and postoperatively, the girl did not have ischemic neurological defects all the time. 

Bacteriologic culture of the excised tissue confirmed a localized staphylococcus aureus infection of the arterial wall. Histopathology of the specimen of the common carotid artery documented an acute purulent inflammation. After this operation the further clinical course was uneventful. Two weeks later the girl was dismissed. 

## 2. Discussion

Thyroglossal duct cysts are the most frequent congenital abnormalities of thyroid glands [1]. In particular, rapid growth of the cystic mass and the presence of a mural nodule on ultrasound, especially with calcifications, could be predictive for cancer arising in thyroglossal duct cysts [2]. Carcinoma is detected in 1% of the resected thyroglossal duct cysts, histopathologically defined as papillary cancer [3]. Today, thyroid gland surgery has a very low incidence of severe complications. In general, thyroid surgery is an aseptic procedure which does not require antibiotic prophylaxis. Septic morbidity plays a subordinate role in the literature of thyroid surgery. Thomusch et al. described complication rates of thyroid surgery in a prospective multicenter study with 7266 patients. 2,7% of the patients had haematoma or haemorrhage and only 0.6% suffered from wound infection [4]. In a multicenter study of over 5 years, Rosato et al. cover postoperative data of 14,934 patients after thyroid surgery. The wound infection rate was only 0.3% [5]. Most ruptures arise from a preexistent inflammation of the carotid artery [6]. Because of their anatomic location, pharyngeal abscesses most frequently involve the internal and less often external carotid artery and favour the carotid bifurcation [6, 7]. Infections are most frequently caused by Staphylococcus aureus. The variety of reported organisms included Streptococcus, Salmonella, Klebsiella, and Escherichia coli. Additionally, the involvement of more gram-negative aerobes and anaerobes is noted [8].

Bleeding is an important intra- and postoperative complication in thyroid surgery with a possibly life-threatening clinical presentation. These bleedings occur almost always within the first 48 hours. The most common cause of post-thyroidectomy haemorrhage was bleeding from the thyroid lobe stump or from a thyroid vessel [9]. Up to now, there is no case described in the literature in which an acute severe artery bleeding was caused by a wound infection 8 days after thyroid surgery. Nakao et al. described two cases of severe bleeding. One patient had a bleeding from the common carotid artery and the other from the brachiocephalic artery after resection and reconstruction of the airway because of advanced thyroid cancer [10]. Machens and Dralle described three patients with a mycotic aneurysm of the common carotid artery induced by infection between 3 and 6 days after a cervical reoperation due to thyroid carcinoma. The diagnosis of mycotic aneurysm was confirmed on histopathology [11]. Neoplasm of the thyroid can also invade the artery. Zhou et al. reported four cases with postoperative rupture of carotid artery invaded by head and neck cancer. The tumours had been taken off from the carotid arteries and the carotid artery remained in situ [12]. This is the first case report with an arterial bleeding caused by a septic arrosion of the common carotid artery after thyroid surgery. In this case, several peculiarities are obvious: the extremely short time between the onset of the inflammation and the rupture of the artery. In our case, the common carotid artery had no aneurysm or neoplasm as a prerequisite for a septic arrosion bleeding. Additionally, the age of the patient excludes preexisting lesions in the vessel like atherosclerosis.

## 3. Conclusion

Septic rupture of the common carotid artery following total thyroidectomy is extremely rare and generally a life-threatening event. Particular attention should be devoted to the condition of the greater vessels. Whenever a wound infection is suspected after a cervical redo operation, a broad opening of the wound should be performed without hesitation. Additionally, the wound infection should be treated by intravenous antibiotics. An aggressive surgical management and a close monitoring of the patient are mandatory. A rapid eradication of the septic focus is crucial to avoid septic bleeding.
